# Phytochemical Composition of Clonally Propagated *Artemisia annua* L. in Different Geographical Locations and Its Commercial Supplement Quality

**DOI:** 10.3390/molecules31111854

**Published:** 2026-05-28

**Authors:** Melissa J. Towler, Joshua J. Kellogg, Pamela J. Weathers

**Affiliations:** 1 Biology and Biotechnology Department, Worcester Polytechnic Institute, Worcester, MA 01609, USA; eeyore@wpi.edu; 2 Department of Veterinary and Biomedical Sciences, Pennsylvania State University, University Park, PA 16802, USA; jjk6146@psu.edu

**Keywords:** *Artemisia annua* L., artemisinin, flavonoids, thin layer chromatography, product quality, mass spectrometry, field cultivation

## Abstract

Global cultivation of *Artemisia annua* L. for the isolation of artemisinin as the current best antimalarial therapeutic mainly takes place on large plantations, but there is an increasing cultivation of the plant for more local use and the supplement market. Phytochemical consistency is a major concern among growers and also regulatory bodies. Long-term cultivation and harvest of field-grown clonal *A. annua* have not been measured for consistency and between different geographical regions. Here, artemisinin and other phytochemicals were measured in clonal *A. annua* grown in two different US geographical locations. Five Florida (FL) and nine Massachusetts (MA) crops were analyzed for artemisinin and flavonoids. TLC (thin layer chromatography) profiles and mass spectrometry analysis were also compared. Artemisinin content dropped by about 10% after transfer from MA to FL, but the flavonoid content increased 2.6-fold. Artemisinin and flavonoid profiles were relatively consistent within each location, but flavonoids differed significantly between the two locations. We also analyzed the artemisinin content of several US commercial *A. annua* and artemisinin supplements. Despite manufacturer claims, about half the samples contained no detectable artemisinin. Together, this study enhances the knowledge about *A. annua* field crops and *Artemisia*/artemisinin supplements being used globally and in the US for therapeutic purposes.

## 1. Introduction

**Figure 1:**
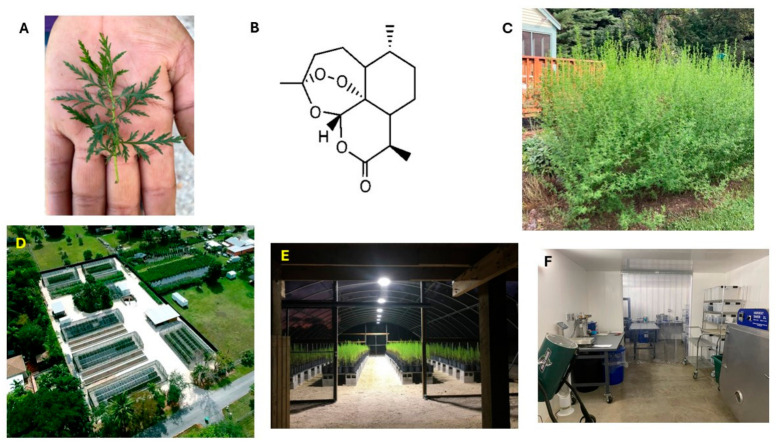
*Artemisia annua* and cultivation. (**A**) *Artemisia annua* L. SAM (a clonal cultivar) cultivar; (**B**) artemisinin (ART); (**C**) *A. annua* field cultivation in Stow, MA; (**D**) Atelier Temenos in Homestead, FL; (**E**) night lighting to inhibit anthesis; (**F**) cGMP (current Good Manufacturing Practice) harvest processing area in Atelier Temenos. (**D**–**F**) are courtesy of Atelier Temenos.

*Artemisia annua* L. (Figure 1) is a globally grown and produced medicinal plant mainly used for the production and extraction of the antimalarial drug artemisinin (ART) (Figure 1B), with an estimated 2026 global market value of US$121.8 million [1]. To improve its bioavailability, ART is derivatized to artesunate, artemether, and dihydroartemisinin and a derivative is combined with another antimalarial drug to yield different versions of artemisinin combination therapy (ACT), the current treatment for malaria.

For over two millennia, the plant has been used to treat various ailments [2] and continues to be used globally as a traditional medicinal tea infusion to treat malaria and other parasites in areas where ACTs are not readily available. Such traditional use is also more accepted by people in low- and middle-income countries. Although the World Health Organization (WHO) has cautioned against the use of such traditional therapies [3], the World Health Assembly (WHA) recently proposed a new strategy to develop more evidence-based science on traditional medicines for their more cost-effective use [4].

A traditional medicine falls within the US Food and Drug Administration’s (FDA) definition of a botanical drug consisting of “…vegetable materials, which may include plant materials, algae, macroscopic fungi, or combinations thereof. A botanical drug product may be available as (but not limited to) a solution (e.g., tea), powder, tablet, capsule, elixir, topical, or injection” [5]. Botanical drugs are not single molecules, but rather complex mixtures being developed for regulatory approval for a variety of ailments. The US FDA reported 739 botanical IND (investigational new drug) applications submitted in 2020 [6]. The development of a botanical drug, however, requires that these complex mixtures remain consistent to yield a reliable therapeutic outcome [7,8,9,10].

Against malaria, the whole plant matrix has been shown to be more effective than ART [11,12], with many of the other effective compounds identified as flavonoids [13,14,15]. Thus, plants with high ART + high flavonoid contents may have added benefits, especially when used *in toto*, e.g., as encapsulated dried leaves, or as a traditional tea infusion.

One of the concerns of the WHO is that there is too much phytochemical variation among *A. annua* plants to provide consistency to ensure reliable and adequate dosing of ART. Although widely grown across temperate and subtropical regions of the world [16,17], its phytochemical content, including ART, can also change during the developmental phases of *A. annua* [18,19,20,21], and in response to many other variables including nutrients [22], seasons [23], water stress [24], and geolocation [25], for example. Thus, it is critical to control as many of these variables as possible.

*A. annua* outcrossing also results in variations in ART and likely other phytochemicals. Self-pollinated plants, however, yielded homozygous progeny with low variation in ART content [26,27]. In another instance, Wetzstein et al. [28] showed that six different clones of *A. annua* produced reasonably consistent levels of ART in two successive annual crops. Based on those studies, we posited that clonally produced *A. annua* crops would also contain consistent phytochemicals and that, even with changes in geography, their ART content may not vary significantly. Here, we show the results of a long-term study that analyzed ART and especially focused on the total flavonoid contents of multiple crops of a clonal cultivar of *A. annua* propagated via rooted cuttings and grown in two different geographical locations in the United States. Because label validation of the contents of herbal medicines is also a major concern [29], we have included an analysis of the ART content of some commercially available supplements of *A. annua* and ART that were or are on the US market. This report is an expanded version of published proceedings of a presentation at the 1st International Symposium on *Artemisia*, Arusha, Tanzania, 10–12 October 2025 [30].

## 2. Results

### 2.1. Artemisinin Content

The ART content among crops within a single geographical area (MA or FL) varied among harvested crops somewhat but remained relatively high and did not decline over time (Table 1). In MA, the ART content ranged from 7.10 to 12.89 mg/g DW (dry weight) and averaged 10.34 mg/g DW of leaf mass for nine annual crop harvests.

In FL, the average ART content ranged from 7.49 to 10.22 mg/g DW and averaged 9.37 mg/g DW for five crop harvests in two years, about 10% less than crops in MA. While there were significant variations among the individual harvested crops in each area (Table 1), there was no indication of a continuing decline or increase in ART content, with the observed variations likely the result of local weather changes during the growing period.

**Figure 2:**
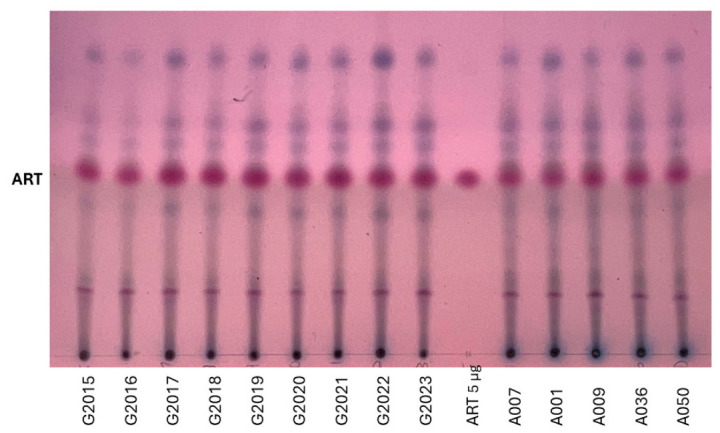
Comparative *p*-anisaldehyde reagent-stained TLC showing profiles of *A. annua* cv. SAM crops grown in Stow, MA, and in Homestead, FL. G2015 to G2023 are the Stow garden crops that correlate with the data in Table 1, followed by artemisinin (ART) 5 µg standard (labeled dark pink spots), then the Homestead, FL, Artecinua™ crops that also correlate with Table 1 from SB#1.007.2022 to SB#1.050.2023. Equal amounts of extract representing 0.5 mg dry mass were applied for all crop samples.

The *p*-anisaldehyde reagent-stained TLC (thin layer chromatography) profiles of the materials listed in Table 1 are shown in Figure 2. The pattern of phytochemicals in each crop was quite consistent with the amount of ART, and was lower, for example, in the Garden 2015 and 2016 crops, as measured by GCMS (gas chromatography–mass spectroscopy). Nevertheless, the MA and FL TLC profiles of compounds stained with *p*-anisaldehyde reagent were similar overall (Figure 2). ART also was relatively consistent between the two locations (Figure 2).

### 2.2. Flavonoid Contents

**Figure 3:**
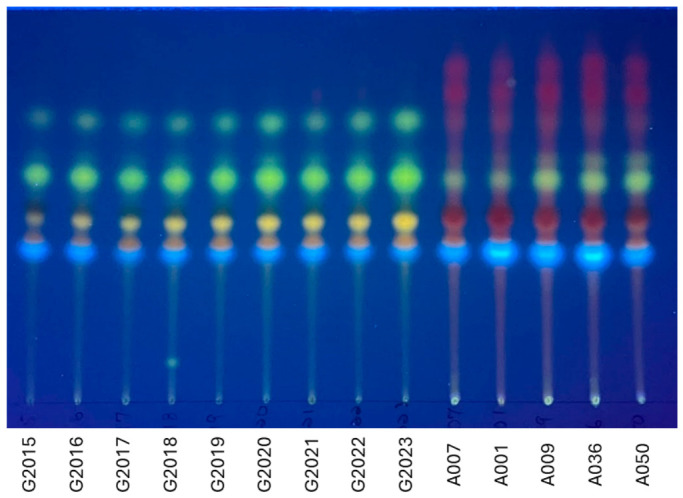
Comparative 2-aminoethyl diphenylborinate-stained TLC showing general flavonoid profiles of *A. annua* cv. SAM crops grown in Stow, MA, and in Homestead, FL. G2015 to G2023 are the Stow garden crops, followed by the Homestead, FL, Artecinua™ crops from SB#1.007.2022 through SB#1.050.2023 that correlate with the data in Table 1. Equal amounts of extract representing 0.5 mg dry mass were applied for all crop samples.

Total flavonoids in the MA crops ranged from 3.27 to 4.30 mg/g DW, with an overall average of 3.74 mg/g DW (Table 1). In FL, however, total flavonoid levels of this clonal cultivar were substantially greater, with contents ranging from 8.46 to 10.62 mg/g DW, with an overall average of 9.76 mg/g DW. The variation among crops in MA was about 23% and in FL was about 20%. Although the TLC flavonoid profiles within each location were consistent, they differed substantially between the two locations (Figure 3). Mass spectrometry analysis of those compounds identified as flavonoids in MA and FL crops are listed in Supplemental Material Table S1.

**Figure 4:**
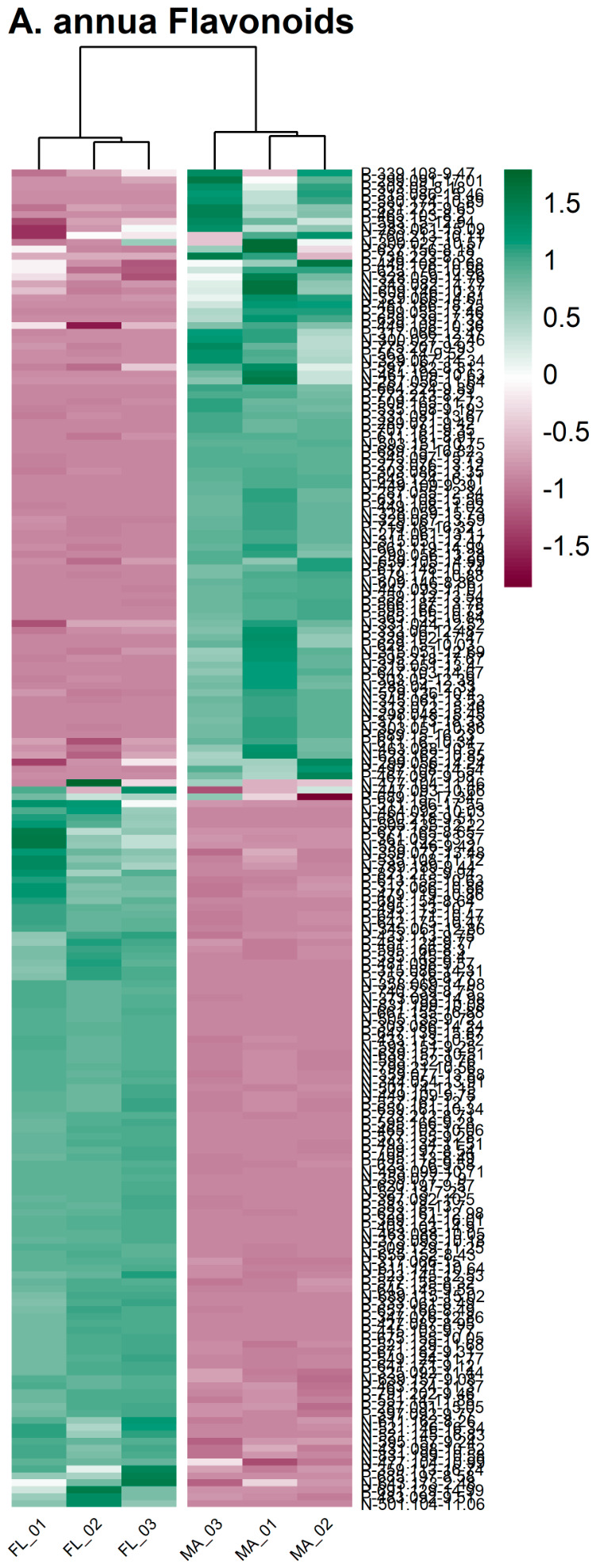
Heatmap showing flavonoid content of samples of clonal *A. annua* grown in MA and in FL.

**Figure 5:**
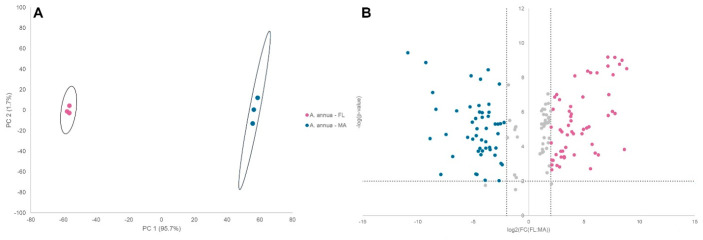
PCA scores plot (**A**) and volcano plot (**B**) of clonal *A. annua* grown in MA and in FL. Lines in the volcano plot represent a cut-off of 2-fold change between the two locations, as well as a statistical significance cutoff of *p* < 0.05. Dots that are gray fall below one or both of these thresholds.

Flavonoids and related structures were determined using CANOPUS (class assignment and ontology prediction using mass spectrometry), a computational tool for chemical class annotation [31]. Flavonoid structures were putatively annotated using available databases where possible, and the top CSI:FingerID hit was taken as the compound identification. Features which could not be putatively identified but were categorized as “flavonoids” or “isoflavonoids” with a confidence score greater than 0.5 were retained in the dataset shown in Table S1. These features were compared against the peak area data from MZmine to tabulate a final dataset, resulting in a total of 189 flavonoid structures used for analysis. The heatmap illustration showed, as expected, some intra-sample variation, but there was greater differentiation in the flavonoid profiles between the samples of clonal plants grown in the two locations (Figure 4) and this is what drives the difference in PCA (principal component analysis) plots (Figure 5A).

Of the 189 probable flavonoid compounds in the MA and FL plants, PCA showed significant differences between the plants grown in the two locations (Figure 5A), with volcano plots noting that there was a substantial number of compounds that did not differ much in content (Figure 5B). There were 23 flavonoid compounds that were originally present in the MA plants but were undetectable in the FL plants (Table S1) and 23 new flavonoids were produced in the FL plants that were not detectable in the MA plants (Table S1). Of the compounds present in the MA plants, 64 increased >2-fold in the FL plants (ranging from 2.02 to 71.85-fold). Another 13 increased only slightly from >1- to 2-fold, after acclimating to the FL environment.

### 2.3. Phytochemical Shift with Geographical Shift

**Figure 6:**
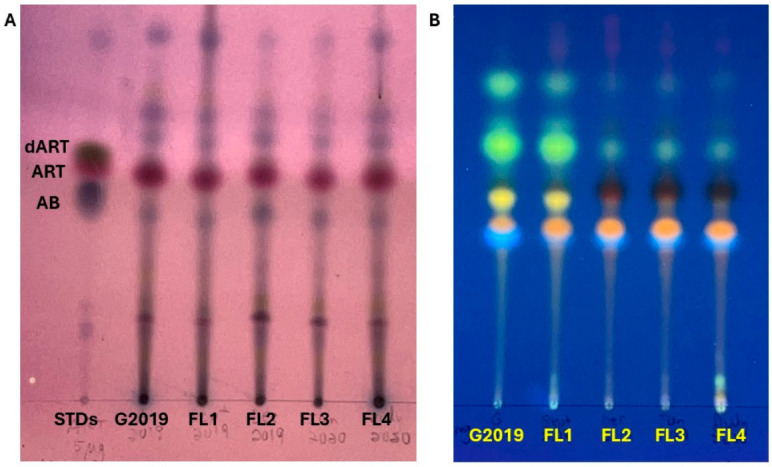
TLC profiles showing transition of phytochemicals after transplanting and subsequent crops of *A. annua* cv. SAM from MA to FL. (**A**) TLC standards are deoxyartemisinin, dART; artemisinin, ART; and arteannuin B, AB. Samples are methylene chloride extracts, representing 0.5 mg leaf DW of G2019, MA field-grown *A. annua* cv. SAM plants grown May–September 2019 for comparison; FL1-FL4, respectively are FL-grown *A. annua* cv. SAM plants from September 2019, December 2019, January 2020, and July 2020; all were stained with *p*-anisaldehyde reagent. (**B**) TLC of the same samples were separately run and stained with 2-aminoethyl diphenylborinate to show general flavonoids and viewed under UV.

The SAM cultivar was shared with Atelier Temenos in 2019, and early phytochemical analyses of ART and other phytochemicals visualized via TLC with *p*-anisaldehyde reagent in the transferred plants were similar to those in MA (Figure 6A) and to the later crops once commercial production began (Figure 2). By 2023, the ART content had stabilized to about 1% of leaf dry mass (Table 1 and Table 2). However, there was a major shift in flavonoid content with each subsequent crop propagated via rooted cuttings of the original plant shoots (Figure 6B, Table 2), with the initial Florida crop (FL1) still reflecting the flavonoid profile of the plants grown in MA (G2019). Once the FL1 plants were rooted (FL2) and grown to harvest, the flavonoid profile changed significantly, remaining consistent in subsequent crops from successively rooted cuttings (Figure 6B, FL3 and FL4). The total flavonoid content of the FL1 to FL2 and FL3 dried leaf harvests increased 1.63-fold. The FL4 leaves were powdered for use in capsules and then shared with us for analysis, and the total flavonoid content increased further (Table 2); nevertheless, the profile remained the same as for FL2 and FL3 (Figure 6B). The company was investigating cultivation regimens using raised beds and/or shade houses, etc. The flavonoid composition changed further, ultimately resulting in the consistent profile shown previously in Figure 3 of five crops harvested in 2022 and 2023.

### 2.4. Other Commercial A. annua and Artemisinin Supplements

**Figure 7:**
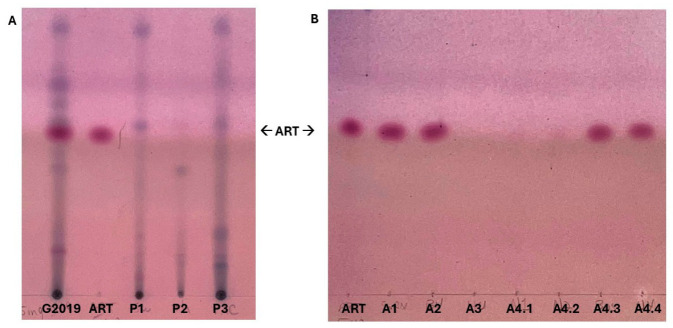
*p*-Anisaldehyde reagent-stained TLC profiles of artemisinin in various commercially available samples of (**A**) G2019 *A. annua* cv. SAM compared to 5 µg of artemisinin standard (ART) and commercial *Artemisia* samples P1–P3; (**B**) shows 5 µg of artemisinin standard followed by the equivalent of 5 µg artemisinin (based on the sample label and weight of capsule contents) of seven commercial artemisinin samples.

During our study, we received for analysis, or purchased, a number of commercially available samples of both ART and *A. annua* plant material being sold on the US supplement market. Of the three dried leaf *A. annua* plant samples we analyzed, P1 and P2 contained a very low amount of ART, a marker compound for *A. annua*, while P3 had undetectable amounts of ART, results further verified via TLC (Figure 7A). The ART estimated via TLC in commercially available capsules for several brand samples was similar to label claims for A1, A2, A4.3, and A4.4, but undetectable in A3, A4.1, and A4.2 (Figure 7B).

## 3. Discussion

The SAM clonal cultivar of *A. annua* was established in MA, then grown in two geographical locations, and harvested as multiple crops over 2–9 years, and its phytochemistry was compared for ART and total flavonoid profiles. To our knowledge, this is the first long-term study of phytochemical ART and flavonoid consistency for a single clonal cultivar of *A. annua* grown in two different locations. Consistency of the clone in each location, however, confirms similar observations by Wetzstein et al. [28]. Once established in a particular location, the phytochemical profile of each crop was quite consistent, with the greatest variation occurring in the flavonoid composition, but mainly after transition to its new location. In contrast, the ART content and *p*-anisaldehyde-stained profile on TLC varied less after the move to FL.

A variety of different environmental stresses impact small-molecule phytochemicals in plants, including drought, biotics, heat and cold, UV index, heavy metals, and salinity [32]; however, identifying which, if any, of these stresses was responsible for changes in the ART or flavonoid contents of the MA plants after the transition to FL was challenging. Drought was ruled out because in both locations water was provided as needed. Biotic stresses, e.g., insect predation, herbivores, or microbial pathogens, were also generally ruled out as none were observed for the harvested crops except as noted below. Indeed, *A. annua* is noted for being somewhat resistant to insect and microbial plant pathogens [33,34]. In MA, there is an overpopulation of deer that regularly forages the area where the plants were grown; however, deer never touched the *A. annua* plants. Indeed, the plants seemed to offer a barrier against deer intrusion. Others have reported the repulsion of deer by *Artemisia* sp. [35,36], with the monoterpene 1,8-cineole reported as one of the most potent deterrents [37]; 1,8-cineole is present in the SAM cultivar used in this study [38]. Rabbits, on the other hand, were occasionally observed nibbling on the lower branches of the plants, so they may have induced more ART production in the MA crops. Rabbits readily consume *A. annua*, possibly for the elimination of intestinal parasites [39]. Temperatures during MA summers are similar to those found among the 12 months in FL, except possibly during the hotter and more humid summer months in FL. Neither location has high salinity or heavy metal contamination.

In a study of 12 main *A. annua* production areas in China, Li et al. [40] noted a positive correlation between both “sunshine time” and high relative humidity (RH) and the ART content of the plants. Another study observed similar results, suggesting a positive correlation between RH and ART for 19 *Artemisia*-growing provinces in China [25]. While RH and sunshine days are challenging to document from the same reliable source, there are some rough estimates available. In the Miami region of FL (~35 mi from Homestead), there are 2943 h/yr of sunshine, with monthly daily averages for the 12-month growing period ranging from 7 to 10 h (~9.41 h). In MA, where the values are available for Boston, there are 2665 h/yr of sunshine days with monthly averages for May–August (the study’s growth period) ranging from 9 to 10 h (~9.31 h/d). The average monthly RHs for MA and FL are about 60 and 57%, respectively. Thus, neither the RH nor the average sunshine days during the MA and FL growth periods of 4 and 12 months, respectively, would seem to account for the 10% greater amount of ART in the MA-grown plants.

UV stress causes plants to produce a myriad of secondary metabolites including alkaloids, phenolics, anthocyanins, hydroxycinnamic acids, terpenoids, and flavonoids [32]. Thus, the greater flavonoid content in the FL-grown plants vs. those grown in MA may be the result of the higher UV index in FL, which is considerably higher on average during the comparable production months in MA (Appendix A Table A1). UVB in particular was reported to increase total flavonoids >2-fold in *A. annua* [41], with the upregulation of key flavonoid genes, including phenylalanine ammonia lyase, chalcone synthase, chalcone isomerase, and cinnamate-4-hydroxylase, possibly through epigenetic demethylation of transcription factor binding sites [42]. UVB also increased ART content about 2-fold and this was associated with the upregulation of several key genes leading to, or involved in, the ART biosynthetic pathway: farnesyl diphosphate synthase, amorphadiene synthase, and artemisinic aldehyde delta-11(13) double-bond reductase [41,43]. That reported increase in ART, however, was in contrast to the 10% decrease in ART that we measured in the FL-grown plants. Taken together, however, there was no obvious stressor that seemed to correlate with the 10% decline in ART in the FL plants.

Total flavonoid content, on the other hand, may have substantially increased in the FL plants as a result of the generally higher UV index in that location. However, 4 of the 12 months have a lower UV index than the 4 months in MA, so the successive crops from that period should have had lower total flavonoids, but they did not. At midday, terrestrial radiation is about 95% UVA (315–400 nm) and 5% UVB (280–315 nm), and the ozone layer removes most UVC (100–280 nm) and UVB [44]. Based on the aforementioned studies, UVB may be the specific stressor within the composite UV index that increased the total flavonoids in the FL plants. Although UV radiation in general has about 5% UVB [44], to our knowledge, there is no direct measurement of the proportion of UVB in either location.

One might expect that the 2.6-fold increase in flavonoids in the FL crops would create a carbon shift and concomitantly decrease ART levels, but that did not occur. A possible reason is that ART and total flavonoids seem to be co-regulated via similar transcription factors in *A. annua* [45,46], so it seems that this SAM cultivar is not compensating for higher total flavonoids by lowering its ART content. Although some have suggested otherwise [47], flavonoids are generally recognized as having synergistic activity with ART, e.g., against the malaria parasite [14,15,48,49].

Having reliable supplements that contain the stated amount of ART is crucial, and counterfeit ART or *Artemisia* products put patients at risk. For example, in Benin, a small study of three commercially available *Artemisia* products showed that one had no detectable ART [50]. While there are literature claims of ART in other *Artemisia* species, amounts are at best substantially lower compared to *A. annua*, especially the cultivars recognized as high ART producers at >1% of DW, i.e., cv. SAM used in this study. In this study, both plant materials (P1–P3) and several of the commercial ART-containing capsules (A1–A4.4) had either no ART or very little. Supplements on the US market are poorly regulated and thus, without validation, a buyer is not assured of reliable content. Indeed, according to the Federal Food, Drug, and Cosmetic Act as amended in 1994 by the Dietary Supplements Health Education Act (DSHEA), it is the responsibility of dietary supplement companies to ensure their products meet the safety standards for dietary supplements and are not otherwise in violation of the law. Thus, without political will, the US supplement industry will continue to sell unreliable material, and this only hurts the entire industry.

## 4. Materials and Methods

### 4.1. Plant Material Cultivation and Harvest

*Artemisia annua* L. cv SAM (voucher MASS 317314) was grown annually for 9 yrs each summer in small garden plots located in Stow, MA (DD coordinates: 42.43704 N, −71.50562 W). Plants were propagated via rooted cuttings from plants grown at Worcester Polytechnic Institute on a windowsill. The rooted cuttings were grown to 10–20 cm, hardened for 2–3 d in partial sun, and then transplanted mid-May in Stow, MA (see Figure 1C for the 2017 crop). Compost was mixed into the soil prior to tilling. No further fertilizer was provided. Plants were watered as needed throughout cultivation. Field harvest was just before 1 September each year, as that is the date when the photoperiod in MA shifts plants into anthesis. In March of 2019, several small, rooted plants (up to 25 cm height) of the SAM cultivar were provided to Atelier Temenos, LLC (Figure 1D), located in Homestead, FL, USA (DD coordinates: 25.46933 N, −80.46749 W), and they proceeded to continue clonal propagation via rooted cuttings to ultimately obtain enough material of consistent quality to market. To compare the ART and general flavonoid contents of the *A. annua* SAM cultivar as it acclimated to the FL conditions, we analyzed the extracts of four crops: September (FL1) and December (FL2) of 2019, and January (FL3) and July (FL4) of 2020. In October of 2021, the material was on the retail market as the supplement, Artecinua™. To maintain plants in the vegetative stage in the FL nursery, overhead light was provided for 30 min each night (Figure 1E). Five crops of Artecinua™ were grown and harvested over approximately two years. The Atelier Temenos *Artemisia* material is annually inspected and certified for current Good Manufacturing Procedures (cGMP). The most recent certification for capsule production was audited on 26 June 2025, by ASI, St. Ann, MO, and both *A. annua* and *A. afra* were given a dietary supplement cGMP certification (ASI certification: latest audit: 26 June 2025, expiration date 26 July 2026). In MA, plants were harvested and dried for about a week in a shaded screened porch, after which they were transferred to the WPI and further dried in a well-ventilated 25 °C room; 3–8 months later, the dried leaves were manually removed and successively processed through a 2 mm brass sieve, then stored in zippered plastic bags until analysis in 2024. The FL crops were harvested and dried by Atelier Temenos (Figure 1F). The five FL commercial crop samples were from harvests in 2022 and 2023: SB#1.007.2022 (A007), SB#1.001.2023 (A001), SB#1.009.2023 (A009), SB#1.036.2023 (A036), and SB#1.050.2023 (A050).

### 4.2. Extraction and Analysis of Artemisinin and Total Flavonoids

Dried leaf samples were extracted using methylene chloride and ART was quantified using gas chromatography–mass spectrometry(GCMS) (see Supplemental Figure S1), as previously described [51], with the addition of a powdering step to 100–150 µm particle size in a flat-blade coffee grinder (KitchenAid coffee grinder Benton Harbor, MI USA BCG111OB) for the MA-grown dried leaves prior to extraction, as previously detailed [52]. Authentic ART (Cayman Chemical, Ann Arbor, MI, USA 11816) was used as a standard. Total flavonoids were quantified from the same extract using a modification of the AlCl_3_ (Sigma-Aldrich, St. Louis, MO, USA) method [53], as detailed in Kane et al. (2022) [51], with results expressed in quercetin (Sigma-Aldrich Q4951) units based on a standard curve.

### 4.3. Commercial Samples

US-sourced commercial samples (P1–P3) of *A. annua* dried leaf material were extracted as already described for the MA and FL leaf material. However, for US-sourced capsules (A1–A4.4) claiming various forms of ART content, the procedure was altered to accommodate the presence of excipients. Sample extraction was complicated by the presence of a number of excipients which included magnesium stearate, rice flour, gelatin, hypromellose, cellulose, leucin, silicon dioxide/silica, and maltodextrin, making GCMS analyses challenging. For those samples, the material was removed from 4 to 6 randomly selected capsules and 25 mg from each was weighed in glass test tubes. Methylene chloride (4 mL) and deionized water (1 mL) were added and then tubes were extracted for 30 min in an ultrasonic water bath. The methylene chloride phase was separated and dried under nitrogen flow. Six random capsules were weighed as a group to measure the average weight per capsule, and again after emptying to determine the net content of the material per capsule. This information was used to calculate the amount of ART per weight of material and then compared to the ART content per capsule claimed on the product label.

### 4.4. Thin Layer Chromatography

Dichloromethane extracts of plants equal to 0.5 mg dry leaf weight were spotted on Si-gel 60 F_254_ plates (EMD Millipore, Burlington, MA, USA cat # 1.05735.0001), run in a mobile phase of 5:11:4 dichloromethane, hexane, and acetone, plus 0.5% *v*/*v* glacial acetic acid, and then sprayed with *p*-anisaldehyde reagent (1 mL glacial acetic acid, 2.5 mL sulfuric acid, 75 mL ethanol, and 2 mL *p*-anisaldehyde (Sigma Aldrich A88107-100G)), heated for 10 min at 105 °C, then viewed under visible light. The visible limit of detection of ART on the plate after staining was 0.5 µg. ART, deoxyartemisinin (Toronto Research Chemicals, Vaughan, ON, Canada TRC-D232150), and arteannuin B (gifted by Nancy Acton, Walter Reed Army Institute of Research, Silver Spring, MD, USA) can be identified by their respective Rf positions and stains of dark pink, brown, and blue, respectively. For commercial samples where the ART content was specified, the amount of extract spotted on the TLC plate was, in theory, equal to 5 µg, as determined from the calculated ART content per weight of material.

For general flavonoids, extracts equal to 0.5 mg dry leaf weight were spotted on Si-gel 60 F_254_ plates and run in a mobile phase of a 10:5:4:1 mixture of hexane, acetone, ethyl acetate, and formic acid, sprayed with 0.5% (weight/volume, *w*/*v*) 2-aminoethyl diphenylborinate (Sigma Aldrich D9754) in methanol, which stains various flavonoids, and viewed under UV light.

### 4.5. Metabolomic Profiling

*A. annua* FL (SB#1.039.2025) and MA (G2023) dried leaf samples were ground to a fine powder prior to extraction, and all extractions were performed in triplicate. The MA samples were MA_01, MA_02, and MA_03; FL samples are FL_01, FL_02, and FL_03 in Figure 4. A 1:1 (g/mL) solid–liquid extraction was performed by combining powdered material with 80% aqueous methanol and 0.1% *v*/*v* formic acid and shaking at 200 rpm at room temperature for 16–18 h. Following incubation, solid material was removed via vacuum filtration with 0.2 μm filter paper and liquid extract was dried to completion via rotary evaporation. Dried extracts were stored at −80 °C until analysis.

Samples were prepared at a dried extract residue concentration of 1 mg/mL in LC-MS-grade methanol with 1 μm chlorpropamide (Santa Cruz Biotechnology, Dallas, TX, USA sc-234350) as an internal standard. Ultra-high-pressure (UP) LC-MS data were acquired using an Orbitrap Fusion Lumos Tribrid mass spectrometer (ThermoFisher Scientific, Waltham, MA, USA) with an electrospray ionization source coupled to a Vanquish UHPLC system (ThermoFisher Scientific). Injections (5 μL) were separated by reverse-phase UPLC using an Acquity BEH C18 column (100 mm × 2.1 mm, 1.7 μm particle size (Waters Corporation, Milford, MA, USA)) held at 55 °C with a flow rate of 100 μL/min. The following binary solvent gradient was employed with solvent A (LC-MS grade water with 0.1% formic acid), and solvent B (LC-MS grade acetonitrile): 0–1 min, 3%B; 1–15 min, 3–15%B; 15–16 min, 15–95%B; 16–18 min, 95%B; 18–18.1 min, 3%B; and 18.1–20 min, 3%B.

Mass spectrometry was conducted using an electrospray ionization source with a positive and negative ion spray voltage of 3500 V/2700 V, respectively, sheath gas pressure of 25 AU, auxiliary gas pressure of 5 AU, ion transfer temperature of 275 °C, and vaporizer temperature of 75 °C. MS^1^ data were acquired with an Orbitrap resolution of 120,000, scan range of 100–1000 Da, and RF lens of 50% in profile mode. MS^2^ data were collected in a data-dependent manner using an intensity threshold of 2.5 × 10^4^ and 60 s dynamic exclusion with a stepped HCD collision energy of 15, 30, and 45%, and a resolution of 7500. Raw spectral data were deposited to The Pennsylvania State University’s ScholarSphere repository (https://scholarsphere.psu.edu/resources/62ebc9cc-a48c-457b-9c27-85ced5b671e8 (accessed on 15 April 2026); doi:10.26207/xnay-0p67).

### 4.6. Data Processing and Annotation

The UPLC-MS/MS data were analyzed and processed using MZmine 4.9.14 [54]. Masses were detected with a noise level of 1 × 10^5^ counts, and chromatograms constructed with 5 minimum consecutive scans, 3 × 10^5^ minimum intensity and 0.005 Da tolerance for *m*/*z* variation. Chromatograms were smoothed with a 5-point Savitzky–Golay algorithm and resolved with a local minimum resolver with a 0.9 chromatographic threshold, 3 × 10^5^ minimum absolute height, 2.5 minimum ratio peak top:edge, peak duration <1.0 min, 5 minimum scans, 0.2 min retention time tolerance, and 0.005 Da *m*/*z* tolerance. Resolved feature lists were deisotoped and joined with a 3:1 *m*/*z*:retention time weighting. Final aligned datasets were blank-subtracted, duplicate-filtered, and gap-filled (0.005 Da *m*/*z* tolerance and 0.12 min retention time tolerance). Features were checked using an RSD filter and only retained if the RSD across the three triplicates was <0.35.

Annotation was carried out using the SIRIUS platform, which integrates multiple advanced algorithms to predict structure (including ZODIAC, CSI:FingerID, COSMIC, and CANOPUS). All MS^2^ data from the two samples were input into SIRIUS and run against the in-house databases, as well as uploaded databases from GNPS [55], LeafBOT [56], MASSBANK [57], and the Vaniya–Fiehn Natural Products Library [58].

### 4.7. Statistical Analysis

For the field-grown plants, leaves were pooled for each harvest, with 5–6 samples extracted and analyzed from each harvest. Commercial products had ≥4 independent samples that were extracted and analyzed ART and total flavonoid contents were statistically analyzed for significant differences using ANOVA. Unsupervised multivariate analysis (principal component analysis) was performed using the cube-root-transformed and Pareto-scaled dataset with the *mdatools* package in R (version 4.5.3).

## 5. Conclusions

To our knowledge this is the first long-term study of a single clonal cultivar of *A. annua* that has been analyzed for its phytochemical consistency over many harvests and within two distant geographical locations. Once established in each location, the phytochemical profile of each crop remained consistent through multiple harvests. Although phytochemical composition changed with geographical location, these results indicated that if plants are propagated clonally, composition will remain consistent in each location. Clonal propagation via rooted cuttings is more labor intensive and thus somewhat more costly; however, the reliability of phytochemical contents may outweigh the greater production cost. While the ART content of plants grown in each location varied somewhat among the harvests and declined by about 10% after the move from MA to FL, it did not vary as much as anticipated. Clearly, it is important to investigate how the ART content of clonally propagated *A. annua* fares wherever the species is grown, as there may be soil or other environmental factors that could alter the ART content in that location. Nevertheless, these results suggest ART content stabilizes if obtained from clonally propagated crops.

While the total flavonoid composition within each location remained relatively consistent, after reaching stable production in FL it increased 2.6-fold. Without further in-depth analyses, there is presently no definitive environmental or biological explanation for the flavonoid changes. Again, it seems that if clonally propagated, the flavonoid contents of plants grown in a particular location can also be reliably characterized.

Analysis of a number of commercially available *A. annua* and ART supplements indicated there is a significant problem with product correlation to label claims. Further analysis of a broader range of samples is warranted to minimize risk to the consumer public. Overall, this study provides further information about the reliability of *A. annua* as a potential botanical drug for therapeutic use.

## Figures and Tables

**Table 1:** Artemisinin and flavonoid contents of multiple crops of *Artemisia annua* cv. SAM.

Sample ID	Artemisinin mg/g DW ± SE	Overall Average	Total Flavonoids mg/g DW ± SE	Overall Average
Stow, MA				
Garden2015	8.69 ± 0.57 c,d	10.34 ± 0.36 m	3.43 ± 0.12 d,e	3.74 ± 0.06 x
Garden2016	7.10 ± 0.25 d		3.40 ± 0.09 e	
Garden2017	11.36 ± 0.92 a,b,c		3.67 ± 0.06 b,e	
Garden2018	10.18 ± 0.56 a,d		3.59 ± 0.04 c,e	
Garden2019	12.19 ± 0.73 a,b		3.82 ± 0.07 b,c,d	
Garden2020	9.38 ± 1.07 b,d		3.27 ± 0.03 e	
Garden2021	12.89 ± 0.88 a		4.30 ± 0.10 a	
Garden2022	10.16 ± 0.50 a,d		3.99 ± 0.13 a,b,c	
Garden2023	11.15 ± 0.68 a,b,c		4.07 ± 0.05 a,b	
Homestead, FL				
Artecinua™ SB#1.007.2022	9.26 ± 0.28 a,b	9.37 ± 0.30 n	10.11 ± 0.09 a	9.76 ± 0.21 y
Artecinua™ SB#1.001.2023	7.49 ± 1.15 b		9.93 ± 0.51 a	
Artecinua™ SB#1.009.2023	9.43 ± 0.18 a,b		10.62 ± 0.19 a	
Artecinua™ SB#1.036.2023	9.98 ± 0.74 a,b		9.66 ± 0.09 a,b	
Artecinua™ SB#1.050.2023	10.22 ± 0.26 a		8.46 ± 0.27 b	

For differences within each location per crop, a–e indicates statistically significant differences over the tested years (*p* ≤ 0.05); m, n indicate statistically significant differences between each geographical location for artemisinin (*p* = 0.04), and x, y for total flavonoids (*p* < 0.001).

**Table 2:** Artemisinin and total flavonoid content of *A. annua* during its transition from MA to FL cultivation. (FL1–FL4 crop samples were measured in the same analysis; *n* = 6 for each sample.)

Plant Sample ID ^1^	Date ^2^	Artemisinin mg/g DW ± SE	Total Flavonoids mg/g DW ± SE
G2019	Sept. 2019	12.19 ± 0.73	3.82 ± 0.07
FL1	Sept. 2019	14.87 ± 0.52	4.81 ± 0.06
FL2	Dec. 2019	15.59 ± 0.24	7.85 ± 0.11
FL3	Jan. 2020	15.31 ± 0.13	7.85 ± 0.10
FL4	July 2020	16.28 ± 0.55	12.03 ± 0.68

^1^ G2019, comparison 2019 field crop from Massachusetts; FL, Florida small batch harvests from *A. annua* being scaled up towards commercial production. ^2^ Date of harvest.

## Data Availability

The original contributions presented in this study are included in the article/Supplementary Material. Further inquiries can be directed to the corresponding author.

## Supplementary Materials

The following supporting information can be downloaded at: https://www.mdpi.com/article/10.3390/molecules31111854/s1, Table S1: *A. annua* MA vs FL flavonoids list. Figure S1: Representative GCMS chromatograms of [A] *Artemisia annua* cv. SAM (Garden2023) and [B] Artecinua™ SB#1.036.2023. Peak 1 = deoxyartemisinin; peak 2 = arteannuin B; peak 3 = artemisinin breakdown product #1; peak 4 = artemisinin breakdown product #2; peak 5 = artemisinin.

## Abbreviations

The following abbreviations are used in this manuscript:

AB	Arteannuin B
ACT	Artemisinin combination therapy
ART	Artemisinin
cGMP	Current Good Manufacturing Practice
dART	Deoxyartemisinin
DW	Dry weight
FDA	Food and Drug Administration
FL	Florida
GCMS	Gas chromatography–mass spectroscopy
IND	Investigational new drug
MA	Massachusetts
PCA	Principal component analysis
RH	Relative humidity
SAM	A clonal cultivar of *A. annua* L.
TLC	Thin layer chromatography
WHA	World Health Administration
WHO	World Health Organization

## Appendix A

**Table A1:** Average monthly temperature and UV index for FL and MA locations for one example year (January–December 2022).

Month	FL (Florida)		MA (Massachusetts)	
	UV Index	Avg °C	UV Index	Avg °C
January	5.3	21	NA	NA
February	7.6	21	NA	NA
March	9.8	22	NA	NA
April	10.9	24	NA	NA
May	11.4	26	8.2	19
June	11.7	27	8.8	25
July	11.8	28	9.1	28
August	11.5	28	8.0	27
September	10.5	28	NA	NA
October	8.4	26	NA	NA
November	6.2	24	NA	NA
December	5.2	21	NA	NA

https://www.uvindextoday.com/usa/florida/miami-dade-county/miami-uv-index/historical-data, last accessed 27 June 2025; https://www.uvindextoday.com/usa/massachusetts/suffolk-county/boston-uv-index/historical-data, last accessed 27 June 2025. NA, not applicable.
