# Supplementary material for: Phytochemical Composition of Clonally Propagated Artemisia annua L. in Different Geographical Locations and Its Commercial Supplement Quality
Source: Molecules. 2026 May 28;31(11):1854. doi: 10.3390/molecules31111854 (PMC13257593; doi:10.3390/molecules31111854)
Supplement: Supplementary file 1 [file molecules-31-01854-s001.zip › Figure S1 GCMS figure Grdn 2023 ASB 1036-2023.pdf]

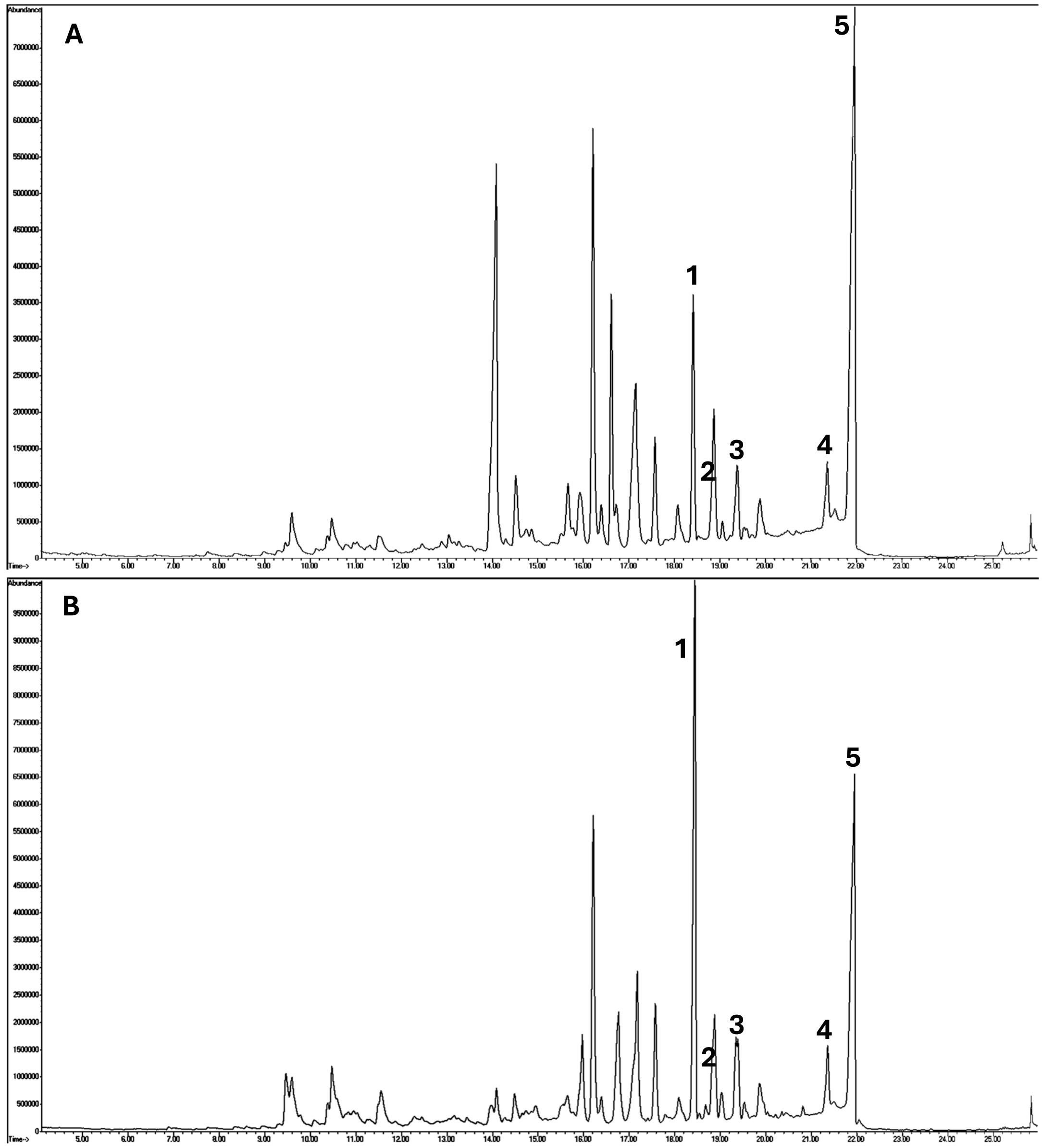

Figure S1. Representative GCMS chromatograms of [A] *Artemisia annua* cv. SAM (Garden2023) and [B] Artecinua™ SB#1.036.2023. Peak 1 = deoxyartemisinin; peak 2 = arteannuin B; peak 3 = artemisinin breakdown product #1; peak 4 = artemisinin breakdown product #2; peak 5 = artemisinin.
